# Hidden in Plain Sight: Lessons From International Case Studies of Child Sexual Abuse in Early Childhood Education and Care Settings

**DOI:** 10.1177/10775595251414844

**Published:** 2026-01-07

**Authors:** Delanie Woodlock, Lenka Olejníková, Michael Salter, Sara Singh, Amy Young, Tyson Whitten, Jon Rouse, Paul Griffiths

**Affiliations:** 1 Childlight (East Asia and Pacific), 7800 University of New South Wales, Sydney, NSW, Australia; 2 Gendered Violence Research Network, 7800 University of New South Wales, Sydney, NSW, Australia

**Keywords:** child sexual abuse, early childhood education and care, child sexual abuse material

## Abstract

Child sexual abuse (CSA) in early childhood education and care (ECEC) settings is sometimes perceived as rare or the subject of a “moral panic”. Recent high-profile cases challenge this characterisation, exposing systemic failures within contemporary childcare environments that enable the sexual abuse of very young children. This article examines six prosecuted CSA cases from high-income countries involving serial offending against children under the age of five to explore how such severe abuse can persist despite regulation and apparent safeguards, and contrary to persistent scholarly claims that child sex offenders do not target ECEC settings. Using gendered organisational theory, our analysis reveals how organisational cultures, gendered power dynamics, and failures in accountability contribute to the occurrence and concealment of abuse. By situating these cases within broader patriarchal structural contexts, the article offers a critical rethinking of institutional responsibility and proposes reforms to strengthen child protection in ECEC settings.

## Introduction

Early childhood education and care (ECEC) settings have long been associated with controversies over child sexual abuse (CSA). While high-profile allegations in the 1980s, such as the McMartin Preschool trial, led to widespread skepticism and claims of “mass hysteria” (Cheit, 2014; Jenkins, 1992), contemporary evidence challenges the view that such abuse is merely the product of moral panic.

Some scholars continue to frame concerns about CSA in ECEC as exaggerated (Munk et al., 2013). Others, such as Parkinson and Cashmore (2017), have argued that ECEC environments are relatively low risk for CSA as compared to those involving older children, claiming that child sex offenders are rarely interested in very young children and there is limited opportunity for abuse. Nonetheless, large-scale offending persists. Recently in Australia, over 1200 children were referred to health authorities after a childcare worker was charged with 70 sex offences (Brown & Higgins, 2025). This case, occurring alongside the prosecution of Australian childcare worker Ashley Griffith (Smee, 2024), exposes a recurring pattern of premeditated exploitation and child sexual abuse material (CSAM) production that mirrors earlier decades (Cheit, 2014), now facilitated by digital technologies.

This study presents a qualitative analysis of six prosecuted cases from high-income countries involving serial offending against children under the age of five. Collectively, these cases document the abuse of at least 245 children. The primary aim is to identify the institutional conditions that enable such abuse and undermine safeguarding mechanisms. Drawing on gendered organisational theory, our analysis seeks to understand how abuse is enabled, concealed, or disrupted by gendered organisational structures and patriarchal cultures (Acker, 1990). The article reviews existing research, outlines our gendered organisational framework, applies this lens to six international case studies, and proposes actionable recommendations for reform.

## Sexual Abuse in ECEC Settings and Early Childhood

Research into child abuse and maltreatment in ECEC settings has been limited and fragmented (Talmon et al., 2023). Few jurisdictions collect or report data specifically on sexual abuse within ECEC contexts (Wonnacott, 2012). A landmark U.S. study conducted in the 1980s provides critical evidence in this regard, identifying 270 daycare centres where 1,639 children experienced substantiated sexual abuse between 1983 and 1985. The abuse was often severe, with 93% of victims subjected to some form of penetrative sexual violence (Finkelhor & Williams, 1988). However, other than this early study, there is an “alarming gap in our knowledge as to the actual rates of daycare maltreatment” (Talmon et al., 2023, p. 521). Studies of children subject to abuse in ECEC settings find significant long-term impacts, including post-traumatic stress disorder, sexualised behaviours and other emotional and behavioural problems, as well as ongoing stress and trauma for their parents (Aviad et al., 2024).

Indicative data on the risk of CSA and exploitation amongst very young children is available from self-report victimisation studies, which consistently show that these children are regularly targeted for CSA. In Australia, 12% of individuals who experienced sexual abuse before the age of 15 reported that the first incident occurred between the ages of 0 and 4 (ABS, 2023). This estimate is consistent with international findings. In the United States, 17% of CSA survivors aged 2 to 17 reported that abuse began between the ages of 0 and 5 (Gewirtz-Meydan & Finkelhor, 2019). Similarly, in Spain, 16% of CSA victims were 5 years or younger at the onset of abuse (Pereda, 2023). These figures are likely to be underestimates, since the sexual abuse of very young children can occur prior to the formation of explicit and declarative memory functions. Hence, victims of sexual abuse at a young age may have no recall of being abused and cannot self-report in victimisation surveys.

In addition to survey and case data, CSAM offers critical evidence of the sexual exploitation of young children. Studies consistently show that prepubescent children are over-represented as victims of highly traded CSAM (C3P, 2016; INTERPOL & ECPAT, 2018). However, pinpointing the proportion of preschool-aged victims is difficult due to inconsistent age categorisation across reporting systems as well as differing methodologies in CSAM data gathering and analysis. For example, INHOPE (2024) reported that in 2024, 93.24% of identified CSAM victims were between the ages of 3 and 13. The Canadian Centre for Child Protection (C3P, 2016) found that 50% of reported CSAM involved children who appeared to be under 8 years old. In contrast, the Internet Watch Foundation (IWF, 2024) estimated that just 3% of detected CSAM depicted children aged 0 to 6. Although CSAM featuring preschool-aged children represents a smaller proportion of overall content, it is disproportionately associated with the most explicit and violent forms of sexual abuse. Evidence across reporting systems indicates a disturbing pattern: the younger the victim, the more likely the material is to involve severe and sadistic abuse (C3P, 2016; IWF, 2024). Given the scale of this risk, research suggests that prevention strategies targeting CSAM offending should be integrated into broader efforts to prevent CSA in institutional settings, including ECEC (Krone et al., 2020).

It is important to consider the organisational context of CSA in ECEC, which provides a unique and complex backdrop to CSA in these institutions. Unlike most of the workforce, the ECEC sector is female-dominated, with as few as 2% of those working in childcare being male (Clarke, 2023). These “pink collar” jobs (Howe, 1977) are usually underpaid and are highly gendered in terms of their tasks, including duties that are deemed stereotypically female, such as caring, cleaning, and nurturing. While females are often disadvantaged when they enter male-dominated workforces, facing what has been dubbed the “glass ceiling,” males in female-dominated workforces do not face similar bias; instead, they can experience the “glass elevator” effect, being promoted to positions of trust and authority (Williams, 1992).

This structural advantage is ideologically sustained by the presumed importance of men. Because care work is culturally devalued, the scarcity of men in ECEC is frequently framed as a deficit that must be corrected, manifesting in a pervasive discourse regarding the need for male role models. Historically, proponents argued that recruiting “brave” men into early education (Plowden, 1967) was necessary to provide children (and boys in particular) with a “surrogate father”. This sentiment persists powerfully in the contemporary sector. Following the 2025 CSA charges against Victorian childcare worker Joshua Brown, male educator Nick Stephens articulated the acute anxiety regarding the scarcity of male staff:

I am the only male role model the children I work with have... the only positive male role model they have... I think without a doubt we're going to lose men, and then recruiting men is harder because that stigma only grows (ABC News, 2025).

Crucially, this fear of “losing men” creates a specific safeguarding risk within ECEC. As Acker (1990) argues, organisations are not gender-neutral; in this context, the desire to retain these “rare” assets grants male staff a “privileged minority status” (Evans, 1997). Institutions may inadvertently provide these men “situational dominance” (Cross & Bagilhole, 2002), thereby insulating them from professional scrutiny to avoid the appearance of bias. This can result in organisational blind spots where motivated offenders can abuse children undetected (Wonnacott, 2012).

## Methodology

This article uses a qualitative case study approach to examine six prosecuted CSA cases from high-income countries (Australia, the Netherlands, the United Kingdom and Sweden). Case selection was based on publicly accessible sources, including court transcripts, official inquiries, and media reports. Cases were included if they involved confirmed criminal prosecution of serial abuse (defined as at least five victims) against children under the age of five within an ECEC setting, and where sufficient documentation existed for institutional analysis. This focus supports a meaningful comparison of institutional risks across jurisdictions with comparable regulatory frameworks. While not exhaustive, these cases reflect serious institutional safeguarding failures and provide transferable lessons for prevention and policy reform.

Case identification followed a multi-pronged strategy. Some cases were located through peer-reviewed literature focused on institutional CSA (e.g. Wonnacott, 2012), which were then cross-referenced with court transcripts, media reports, and official inquiries. Others were identified through systematic searches of news archives and public databases using keywords related to CSA in ECEC settings (combinations of terms such as ‘childcare abuse,’ ‘ECEC sexual abuse,’ ‘early childhood educator convicted,’ and ‘nursery worker CSA’). Each case was corroborated through multiple sources where available, including confirmation from two co-authors (Griffiths and Rouse) with law enforcement backgrounds, both of whom are former members of online CSA investigation teams. This triangulation ensured accuracy and depth of institutional detail.

A structured analytical framework guided the analysis. The primary analytic lens was gendered organisational theory (Acker, 1990). While ECEC is a female-dominated sector, our analysis posits that gendered hierarchies persist, often conferring a privileged minority status (Evans, 1997) upon male staff. We applied a deductive thematic analysis (Braun & Clarke, 2006) to the case documents, utilizing Acker’s (1990) framework of gendered substructures to interrogate the evidence. Specifically, we analysed the records for instances of:

- Cultural symbols: The deployment of the male role model discourse or maternal ideal to justify differential treatment.
- Workplace interactions: Power dynamics that silenced whistleblowers or shielded male staff from scrutiny.
- Organisational logic: Hiring and vetting practices that appeared neutral but structurally privileged male applicants.

Since this study relies on retrospective criminal justice data (court transcripts, inquiry reports, and sentencing remarks) rather than ethnographic observation, we focused on how these gendered substructures were documented in the official records.

There are important limitations to this case study method. In line with established case study design (Flyvbjerg, 2006; Yin, 2018), the goal is not statistical representativeness but the development of in-depth, contextualised insights. Case selection was constrained by the availability of publicly documented material, limiting the scope of the findings. Crucially, this study relies on adjudicated cases where the offender was caught and convicted. This inherently limits our analysis to those offenders who were detected, often through external CSAM investigations, and likely excludes offenders who more successfully evade detection.

## Case Study Analysis

This case study analysis draws on six contemporary, prosecuted cases of CSA in ECEC settings, spanning the period from 2009 to 2024. Each case was selected based on its alignment with the study’s inclusion criteria and capacity to reveal broader institutional patterns. By analysing these cases through the lens of gendered organisational theory, the study identifies recurring dynamics that allowed abuse to persist across varied settings. A detailed account of each case follows, highlighting the organisational conditions that facilitated harm.

### Vanessa George (Plymouth, U.K., 2009)

In 2009, Vanessa George, a 39-year-old nursery worker at Little Ted’s Nursery in Plymouth, England, pleaded guilty to multiple child sexual abuse offences, including seven counts of sexual assault and six counts of creating and distributing indecent images of children (BBC News, 2009). George was suspected of abusing up to 30 children aged between 2 and 5, although the actual number remains unknown due to her deliberate concealment of victims’ identities. Her abuse came to light during an investigation into another offender, Colin Blanchard, whose computer contained CSAM involving infants and toddlers. Police uncovered messages and images exchanged between Blanchard and George, ultimately revealing a network of four women and Blanchard engaged in abuse and image-sharing (Kay, 2009).

Although George was widely described by parents as warm and caring (Morris, 2009), staff later reported that she had begun exhibiting sexually explicit behaviour and conversations in the months leading up to her arrest. This discrepancy illustrates the power of Acker’s (1990) cultural symbols in shaping risk perception. The perception of George as the “nurturing mother” served as camouflage, rendering her predatory behaviour invisible to a community conditioned to view women as inherently safe. George’s close personal relationship with the nursery manager, whose children she babysat, discouraged staff from raising concerns (Wonnacott, 2012). Most of the abuse occurred during routine caregiving tasks, such as nappy changes, when George had unsupervised access to very young children. During these moments, she used her mobile phone to take images and, in at least one instance, brought a sex toy into the nursery to commit abuse (Morris & Carter, 2009).

Structural weaknesses at the nursery enabled George’s offending. She was hired without an interview or reference checks, and oversight by the board of trustees was minimal. Parents had no formal avenues to raise concerns, and staff described feeling powerless to challenge inappropriate behaviour due to a toxic culture of loyalty and fear (Wonnacott, 2012). The abuse caused lasting harm to children, families, and the broader community. George refused to name the children she had abused; however, police issued behavioural indicators of abuse to assist concerned parents. One parent shared, “My children meet all those criteria… They are withdrawn … wetting themselves… I am devastated” (Morris & Carter, 2009). Little Ted’s Nursery was subsequently closed, and a Serious Case Review identified significant regulatory failures.

### Robert M. (Netherlands, 2010)

Robert M. was a 27-year-old daycare worker and babysitter in Amsterdam who sexually abused at least 87 children, all under the age of 4 (Lindauer et al., 2014). The majority of victims were male, and the abuse took place across multiple ECEC centres and private homes. His partner, Richard van O., assisted in concealing the offending. Robert M. specifically targeted preverbal children and reportedly stopped offending once children began to speak. He chose workplaces without CCTV and held positions across several sites, taking advantage of poor coordination between employers (BBC News, 2010).

Institutional factors played a significant role in enabling the abuse. At the time, the Netherlands lacked a centralised vetting system for childcare workers, and services were not required to ensure co-supervision or transparent physical environments (Turrell, 2023). A concern raised by a mother prior to his arrest in 2010 was dismissed by both the ECEC provider and the local police (Pascoe, 2010). This dismissal reflects gendered workplace interactions (Acker, 1990) wherein the institutional hierarchy privileged the male worker’s professional status over the mother’s concerns, effectively silencing the primary whistleblower. Additionally, Robert M.'s earlier conviction for possession of CSAM in Germany in 2003 was not identified during the employment screening process due to failures in international information sharing.

The abuse was detected only as part of an international CSAM investigation. A distinctive toy seen in abuse footage prompted investigators to release images to the public, which eventually led to his identification (Tiemoerie, 2013). Because all the victims were non-verbal, none had disclosed the abuse. In response, Dutch authorities introduced the “Four Eyes Principle,” which mandates that no worker be left alone with a child. Additional reforms included stronger vetting protocols, environmental redesigns to enhance visibility, and long-term support for affected children and families (Turrell, 2023).

### Shannon McCoole (South Australia, Australia, 2014)

Shannon McCoole worked across multiple ECEC settings before taking a position in South Australia’s state-run residential care system (Prosser, 2016). In 2014, he was arrested following a global investigation led by Queensland’s Taskforce Argos, which targets online child sexual exploitation. McCoole was found to have produced and distributed CSAM and had at least seven identified victims aged between 18 months and 7 years, most of whom were female. His offending spanned multiple institutions and extended across international jurisdictions, including Canada and the United States (Prosser, 2016).

McCoole targeted preverbal, disabled, and highly vulnerable children, including those in out-of-home care. He actively sought roles that enabled access to children with limited capacity for disclosure and admitted to receiving little formal training, raising serious concerns about the adequacy of staff screening and professional oversight. Despite a psychological screening report classifying him as “high risk” and “very unsuitable” for working with children, McCoole was employed in multiple frontline roles (Prosser, 2016). He was initially hired by a friend to work in out-of-school-hours care, and behavioural concerns were raised by staff as early as 2010. However, no meaningful action followed, and complaints across agencies were either dismissed or minimised.

A female colleague who raised concerns faced significant workplace retaliation. She reported 18 incidents of intimidation and victimisation following her disclosures (Lee, 2019). Her superiors promoted McCoole to her supervisor, dismissing her and her colleagues as “a bunch of scorned old women.” One senior staff member reportedly described her as “a fucking bitch” who was simply “jealous” that McCoole had been promoted (Lee, 2019). This rapid promotion of a less-qualified male over experienced female staff exemplifies the glass escalator effect (Williams, 1992). These interactions also highlight Acker’s (1990) concept of gendered workplace interactions, in which, by reframing professional safeguarding concerns as women’s jealousy or hysteria, the institution successfully silenced the female whistleblower and protected the higher-status male employee.

In response to McCoole’s arrest, the South Australian government convened the Child Protection Systems Royal Commission (Clarke, 2023). The inquiry led to wide-ranging reforms in recruitment, staff training, reporting protocols, and oversight mechanisms.

### D.N. (Kristianstad, Sweden, 2015)

In 2015, D.N., a 40-year-old childcare worker in Kristianstad, Sweden, was arrested following the sexual assault of a 10-year-old girl at a public amusement park (The Local, 2015). Subsequent investigations uncovered significant illegal material at his residence, including content created during his shifts in preschools throughout Sweden. Authorities discovered that D.N. had been employed through a staffing agency, allowing him to rotate across more than 40 childcare centres (Kristianstad District Court, 2015). He was sentenced for abusing 18 children in his care, who were mainly preverbal girls aged between 12 months and three years, and the sexual abuse occurred during routine caregiving activities, particularly changing nappies (Kristianstad District Court, 2015). Court records emphasized that D.N. expoited a “special trust” and he was described as well liked, experienced, and trustworthy, with no prior reports or complaints regarding his conduct in ECEC environments (Kristianstad District Court, 2015). This unearned trust illustrates the power of gendered cultural symbols (Acker, 1990). Despite his transient status, D.N.’s presentation as “trustworthy” allowed him to access a “patriarchal dividend” (Connell, 1995) of presumed competence that shielded him from the scrutiny that should be typically applied to temporary staff.

A key factor enabling the offending was the structure of the staffing model itself. D.N.’s employment through a temporary agency meant he was often placed in services with little continuity or oversight. Each centre relied on the assumption that the agency had carried out appropriate background checks, leading to a diffusion of responsibility (Kristianstad District Court, 2015). This approach meant behavioural patterns or concerns could not be easily tracked across institutions. As a temporary worker, D.N. was less well-known to permanent staff and was often permitted to carry out caregiving duties alone or under minimal supervision. This fragmentation exposes a flaw in organisational logic, which relied on the myth of the “abstract worker” (Acker, 1990). By treating staff as interchangeable, disembodied units of labour, the system obscured the reality that workers are, in fact, deeply sexed and gendered. This false neutrality allowed the agency to overlook the specific risks associated with granting a temporary, unvetted male access to the intimate care of preverbal children.

### Timothy Luke Doyle (NSW, Australia, 2020)

Timothy Doyle worked as an early childhood educator in New South Wales, Australia, between 2017 and 2019. In late 2018, a mother reported that her child had disclosed sexual abuse by Doyle (McKinnell, 2024). The service issued a prohibition notice following an internal investigation, prompting his resignation. However, there is no public evidence that the matter was reported to police or statutory child protection authorities. This response exemplifies a defective organisational logic (Acker, 1990). The institution framed the abuse allegation as an internal employment matter to be resolved through resignation, prioritizing reputation management over the mandatory legal reporting required to protect children. Over a year later, Australian Federal Police uncovered a USB containing CSAM during a separate investigation, leading to Doyle’s identification as a central figure in an international offender network (Collins & Elsworthy, 2020). He was arrested in June 2020 and later pleaded guilty to more than 300 charges. Sixteen of his 30 identified victims were children in his care at the service (McKinnell, 2024). His former partner, Steven Garrad, aged 22 at the time, was also arrested and convicted of over 120 child abuse offences. The court noted that their relationship contributed to the escalation and persistence of the abuse (McKinnell, 2024).

Doyle exploited routine caregiving tasks to access young children, specifically nappy changing and settling children for sleep, using his trusted position to offend without attracting suspicion (McKinnell, 2024). He recorded and shared material via encrypted platforms, and his involvement in an online network served to reinforce and escalate his behaviour. Although the service took internal disciplinary action, its failure to escalate the mother’s report allowed the abuse to continue for over a year (Murray, 2024).

### Ashley Paul Griffith (Queensland, Australia, 2024)

Ashley Griffith was employed across multiple ECEC settings in Queensland, Australia, for nearly two decades. In 2024, he was sentenced to life imprisonment after pleading guilty to 307 offences against 73 young girls, including rape, indecent treatment, and the production and distribution of CSAM (The King v Griffith, 2024). Griffith systematically targeted females between the ages of two and five and abused them during routine caregiving activities, such as nappy changes and while children were sleeping. Over 19 years, he filmed more than 4,500 images and videos of abuse, which he concealed using encrypted devices. He moved across multiple childcare centres and geographic regions, including time overseas, and cultivated a reputation as calm, caring, and professionally dependable (Antrobus & Brennan, 2024). He was also embedded in online offender networks that encouraged and normalised CSAM production and distribution.

Although concerns were raised about his behaviour, including a mother’s complaint to a service director and a female colleague’s report to the police, no effective action was taken (Antrobus & Brennan, 2024). The abuse was uncovered during an investigation into online CSAM, which Griffith had filmed while employed at his most recent childcare centre. Investigators were able to trace items visible in the footage to a specific provider, ultimately leading to his identification.

Witness statements highlight that Griffith was a trusted and beloved educator. One survivor said: “I wish I were filled with hate, but there is still part of me that thinks ‘that is my favourite teacher’ and that is probably the worst thing.” Another shared: “I had unlimited trust in Ashley…” A young woman who was abused more than 50 times reflected: “I will never know what my life could have been like… I have missed out on a normal childhood” (Smee, 2024). Parents echoed these sentiments of both loss and betrayal. One mother described attending a “stranger danger” event taught by Griffith, noting: “How were we to know the greatest danger to our child was entrusted with her care?” (Smee, 2024). This exploitation of trust exemplifies the risk of gendered cultural symbols (Acker, 1990). By embodying the archetypal “protective male” and leading safety initiatives, Griffith weaponised the sector’s desire for male role models to create a camouflage so effective that even whilst teaching about danger, he remained above suspicion.

The case also involved significant institutional retaliation against whistleblowers. In 2023, former childcare manager Yolanda Borucki publicly disclosed concerns about Griffith after witnessing him “kissing” a young child in a play area (Brennan, 2024). Rather than being supported, Borucki was charged with computer hacking after allegedly forwarding internal documents to her personal account and the media. This punitive response illustrates Acker’s (1990) analysis of gendered workplace interactions. The institution mobilised its resources not to investigate the male authority figure, but to discipline the female whistleblower, prioritising reputation management over child safety in a clear act of institutional betrayal (Smith & Freyd, 2014). The charges were dismissed when the court found insufficient evidence that she had accessed the data illegally or caused provable harm (Brennan, 2024).

## Key Findings and Recommendations

Striking similarities characterise the six international cases spanning more than two decades: primarily male offenders, leveraging the cultural symbol of the male role model, systematically targeted preverbal children, evaded detection, and were ultimately exposed only through external investigations, most often related to the possession and distribution of CSAM. The fact that none of these offenders were uncovered through internal safeguarding systems raises urgent questions about the effectiveness of current child protection frameworks in ECEC environments and points to a systemic pattern of institutional betrayal (Smith & Freyd, 2014).

This study identified several recurring themes that remained consistent across time and geographic contexts. Applying a gendered organisational lens, we highlight the roles of (1) offender behaviour patterns, (2) the mechanics of institutional grooming, and (3) gendered organisational substructures in enabling the sexual abuse of children in ECEC settings. The remainder of this section explores each of the three patterns and their sub-themes in depth, and, based on the findings, offers recommendations for strengthening child protection.

### Offender Behaviour Patterns

#### Offender Networks and the Lone Offender Myth

These case studies challenge the prevailing view of CSA offenders as isolated individuals, revealing instead the existence of networks that facilitate the sharing of CSAM, the exchange of strategies, and mutual encouragement. The evidence in these cases demonstrates that offenders commonly operate within online communities, facilitated and camouflaged by encryption technologies, that normalise and reinforce abusive behaviour and share CSAM of children in their care. McCoole, for example, was a central figure in The Love Zone, a forum on Tor hidden services (the so-called “dark web”) dedicated to the exchange of CSAM, which also included Griffith (Staff Writers, 2023). McCoole later had his sentence reduced for assisting international investigations, including cases in Denmark (Opie, 2018). Once dismissed as the product of moral panic, claims about organised CSA offending have now been substantiated through forensic evidence (Salter, 2012). Indeed, organised offending has been a consistent theme in allegations and investigations of CSA in ECEC for over forty years (Cheit, 2014). However, the proposition that ECEC may be targeted by organised offender networks for the purpose of CSA has not been taken seriously in child protection frameworks and measures. This oversight can be understood through Acker’s (1990) concept of cultural symbols. The persistence of the “lone offender” myth serves a protective function for the institution; it allows ECEC providers to treat abuse as the result of a singular “bad apple” rather than interrogating how the gendered structure of the workforce might attract and shield organised abuse networks.

#### Targeting of Preverbal Children

Across all six case studies, offenders deliberately targeted very young children, exploiting their developmental limitations to reduce the risk of detection. This is evident in the fact that, despite the large number of victims, only one child disclosed abuse (by Doyle). In the case of Robert M., this behaviour was particularly calculated as he exclusively abused preverbal children and stopped the abuse once they began to speak more clearly. In their experience of medical assessment of sexually abused babies and toddlers, Romano and Hayez (2023) identified a highly instrumental type of perpetrator who engages in the serious abuse of children under the age of three, often as part of a larger network of offenders. Such a motivated and manipulative offender is capable of neutralising or co-opting safeguards. These offenders often target very young children due to their limited communication skills, which hinder their ability to give reliable witness statements needed for legal proceedings. This makes ECEC settings “ideal targets for paedophiles” (Briggs, 2014, p. 1415).

### The Mechanics of Institutional Grooming

#### Internal Safeguarding Failures

In each case study, internal safeguarding mechanisms within the ECEC context failed to detect or respond adequately to CSA, and the abuse was discovered only after CSAM investigations or, in one instance, sexual assault of a child in public. As seen in the cases of Griffith, Doyle, George, McCoole, and Robert M., it was the discovery of CSAM that compelled institutions and authorities to take action. In the case of D.N. in Sweden, abuse only surfaced after a separate police investigation unrelated to institutional safeguards. Some institutions repeatedly dismissed or downplayed concerns raised by mothers and female staff, until external, undeniable evidence emerged. This systemic dismissal of female voices points to gendered workplace interactions (Acker, 1990), where the professional status of the male worker is privileged over the evidence provided by mothers and female colleagues. These patterns underscore how institutional responses often depend on proof rather than trust, suggesting that ECEC safeguarding systems may be structured to respond only when abuse becomes irrefutable, rather than working to prevent it.

#### Tolerance for Grooming Behaviour

Most of the offenders in the six cases were well-regarded by colleagues, trusted by families, and liked by the children in their care. Simply presenting as professional and caring often proved sufficient to gain the trust of colleagues, management, and parents, which often continued even after concerns were raised. In some instances, offenders socialised with management and parents, offered private babysitting, and were welcomed into families’ homes. For example, sentencing remarks in the case of Griffith noted that he was often cruel and mocking towards children during the abuse, as revealed in CSAM he produced (The King v Griffith, 2024). Despite this, he was invited to children’s birthday parties and formed close relationships with their families. The case studies corroborate previous research on how offenders often actively groom not only children, but also co-workers, parents, and the institution itself (Briggs, 2014).

Grooming generally refers to how offenders build trust and manipulate those around a child to maximise their opportunities for abuse while minimising the risk of detection (Erooga et al., 2020). “Professional” perpetrators who pursue child-focused employment in order to abuse children typically groom and manipulate institutional hierarchies, taking advantage of “informal” as well as formal aspects of workplace culture and practice (Colton et al., 2010). McAlinden (2006) described how sex offenders engage in “institutional grooming” and manipulate institutions such as the criminal justice system and other organisations into believing they pose no risk to children. Our analysis suggests that this institutional grooming (McAlinden, 2006) relies heavily on the weaponisation of Acker’s (1990) cultural symbols. In this sense, offenders do not just build trust, but also perform specific gendered roles, such as the “surrogate father” or the “maternal carer”. Research also indicates that the general public struggles to recognise behaviours associated with grooming (Winters & Jeglic, 2016). Understanding the use of institutional grooming by offenders is therefore essential for strengthening the protection of children from abuse.

#### Poor Oversight and Cross-Institutional Tracking

The case studies demonstrated how weak regulatory structures, including a lack of oversight of staffing and hiring practices, enabled offenders to move between services without adequate scrutiny or shared information. In a context where child safety should be the central concern, these failures in information sharing and employment vetting reflect a lack of institutional accountability. Across the case studies, offenders gained access to ECEC roles through informal networks, as in the case of McCoole, or without standard hiring procedures, as was the case with George. In other cases, long professional experience led to misplaced trust. Even when concerns were formally raised, such as McCoole being deemed unsuitable to work with children, this did not prevent further employment with children. These vetting failures expose a dangerous organisational logic (Acker, 1990). By treating applicants as abstract, gender-neutral units of labour, the system failed to account for the specific risk profiles associated with the glass escalator phenomenon, where scrutiny was lowered to facilitate the rapid entry of men into the workforce.

In Robert M.’s case, his prior conviction for CSAM offences in another country was not identified during his recruitment, exposing critical gaps in international information sharing. Despite the high vulnerability of children in ECEC settings, there was no coordinated system to monitor individuals’ employment histories or flag prior concerns across institutions and jurisdictions. Offenders were able to transition between services and locations without detection. These examples highlight not only regulatory failures but also a broader disregard for child protection in environments where vigilance should be paramount.

### Gendered Organisational Substructures

The dismissal of whistleblowing is a central marker of institutional betrayal (Smith & Freyd, 2014). Across several case studies, this betrayal was especially acute for women who raised alarms about suspected CSA. In some cases, women’s concerns were not only ignored but actively punished. In the cases involving McCoole and Griffith, female co-workers who voiced concerns were subjected to verbal abuse, demotions, and even formal charges. Their experiences reflect what Ahern (2018) described as “whistle-blower gaslighting”: a tactic where institutions respond to valid concerns with emotional manipulation, isolation, and retaliatory actions designed to erode the whistle-blower’s confidence, credibility, and mental health. Our analysis using Acker’s (1990) framework reveals that this retaliation is not random but rooted in gendered workplace interactions and patriarchal hierarchies. Many of these women were long-serving professionals acting in accordance with their safeguarding responsibilities, yet they were met with severe reprisals. Mothers who raised concerns about their children’s behaviours and physical evidence of abuse were also dismissed. However, research has shown that children are most likely to disclose CSA that occurs in institutional settings to their mothers (Quadara, 2016). The systemic failure to take maternal reports seriously signals a broader devaluation of women’s voices, both as professionals and as parents. This pattern illustrates how gendered organisational structures silence those most likely to detect harm, resulting in a profound institutional betrayal of the primary caregivers.

There is growing consensus that organisational culture is critical to the prevention of institutional sexual abuse, including implicit relations of power and trust based on gender and other factors (Higgins & Moore, 2019). Gendered organisational theory provides a useful framework for understanding the sex-based dynamics of these institutional responses to CSA. Specifically, our findings suggest a perfect storm of gendered blindness. The male role model discourse encouraged institutions to view male staff as precious commodities to be protected. At the same time, the fear of appearing discriminatory, specifically the anxiety regarding false allegations (Munk et al., 2013), silenced female colleagues who might otherwise have raised the alarm. Although ECEC sectors are sometimes accused of being overly suspicious of male staff (McDonald et al., 2024; Munk et al., 2013), evidence shows that men are frequently afforded a privileged minority status (Evans, 1997) in these environments. The male offenders in these case studies likely took advantage of this dynamic.

On the other hand, the case of Vanessa George illustrates how female offenders can exploit gendered cultural symbols that depict women as inherently nurturing and therefore incapable of severe child abuse. Wonnacott (2012), who participated in the inquiry into George’s case, observed that the fact that she was female and the mother of three played a significant role in both facilitating the abuse and suppressing concerns among staff. Although women comprise a small minority of child sexual abuse offenders, the reluctance to view women as potential perpetrators can also contribute to such abuse going undetected. This case reinforces broader research findings that female perpetrators are often perceived as less threatening and may offend alongside male partners, which can obscure their individual culpability (Bensel & Raptopoulos, 2025).

### Recommendations

To address the institutional vulnerabilities identified across the case studies, safeguarding practices in ECEC must move beyond dependence on victim disclosure or post-investigative evidence. Since children under five may not be developmentally capable of reporting abuse, safeguards must be proactive and preventative. We outline five key areas where reforms are urgently needed.

#### Physical and Operational Safeguards

Safeguards must account for the developmental limitations of preverbal children by ensuring that prevention and detection do not depend on victim disclosure. Surveillance measures such as CCTV installation in most areas of the centre, while maintaining privacy standards, can aid both in deterring abuse and supporting investigations. ECEC centres should implement the “four-eyes” principle, requiring at least two adults to be present during nappy changes and other care tasks. A strict no-phone policy could also reduce the risk of image-based offences. Moreover, internal reporting mechanisms should be clearly defined and effectively implemented to ensure that concerns regarding educator behaviour are promptly investigated and addressed.

However, reliance on surveillance must not breed complacency. As noted in the case of Robert M, technical fixes are insufficient if the organisational culture does not support vigilance. There is a risk that CCTV may create a false sense of security, masking the need for rigorous interactional monitoring. Furthermore, operational changes must address specific high-risk routines identified in our analysis, such as sleep times. Recent regulatory changes in Queensland, Australia, removing ‘rest-time’ provisions to ensure active supervision during sleep, represent a positive step towards recognising these specific situational risks (Alston, 2025).

#### Workforce Screening and Grooming Prevention

Stronger staff vetting procedures are required to reduce risks associated with predatory behaviours. Many “working with children” checks rely primarily on criminal history records, which are insufficient for identifying individuals who have not yet come to the attention of law enforcement or who have evaded detection. Therefore, screening processes should be expanded to include validated personality assessments that can identify traits associated with grooming and interpersonal manipulation (Nicol et al., 2022). These assessments may be particularly relevant in detecting risk factors in individuals who maintain a facade of competence and trustworthiness. However, psychometric screening should serve as a supplement, not a substitute, for human vigilance. Over-reliance on screening tools risks a ‘tick-box’ approach to safety that may fail to identify manipulative offenders who understand how to game the system.

Crucially, these tools must be implemented with an awareness of the pink-collar paradox identified in our analysis. Recruitment panels must be trained to recognise the glass escalator bias, ensuring that the desire to increase male workforce participation does not lead to expedited vetting for male applicants. Additionally, staff employment histories should be carefully reviewed, particularly when patterns of frequent job changes or unexplained departures are evident.

Safeguarding training should also explicitly address professional boundaries and institutional grooming. This must move beyond general warnings to address the specific gendered dynamics of privileged minority status (Evans, 1997), empowering staff to challenge the male role model halo when behavioural red flags appear. This includes the importance of maintaining clear, formal relationships with families and avoiding informal or socialised interactions with parents or carers. These standards should be clearly articulated in codes of conduct and regularly enforced, while simultaneously encouraging a culture of open professional collaboration among staff to ensure effective peer monitoring.

#### Digital Investigation and Policy

A consistent pattern across the cases was the role of digital forensic investigations in detecting abuse, particularly through the identification of CSAM. Units such as Taskforce Argos in Australia illustrate how specialised law enforcement teams can identify victims through proactive online investigations. However, these capabilities are not evenly distributed globally, and the growing use of encrypted technologies increasingly challenges their effectiveness. For instance, the National Center for Missing & Exploited Children (2025) reported a significant decline in CSAM referrals between 2023 and 2024, coinciding with the widespread implementation of end-to-end encryption. Policymakers must strike a balance between privacy rights and child protection imperatives and hold technology platforms accountable for developing tools that put children at significant risk.

#### Institutional Courage

The case studies demonstrate that institutional betrayal is an important correlate of institutional child sexual abuse, acting as a risk factor and harm amplifier for children subject to CSA in ECEC as well as their families. Freyd and Becker-Blease (2024) have conceptualised “institutional courage” as the antidote or solution to institutional betrayal. They explained institutional courage in terms of strategies that intentionally foster accountability, transparency, justice, and reparation as a preventative measure against institutional betrayal. Core to institutional courage is recognition of the courage of abuse victims and whistleblowers for speaking out.

Our analysis reveals that the silencing of female whistleblowers, often dismissed as jealous or hysterical, is a key mechanism of institutional betrayal. Therefore, ECEC policies, procedures, and training should be reviewed to instill institutional courage into the practices and culture of the ECEC setting. This requires specific protocols to protect whistleblowers from the gendered retaliation observed in the McCoole and Griffith cases. Institutions and oversight authorities must ensure that proactive efforts to disrupt and prevent abuse are incentivised and rewarded.

#### Economic Constraints and Gendered Labour

Many of these recommendations will not be achievable without addressing the economic and gendered foundations of the ECEC sector. Chronic underinvestment, workforce casualisation, and low pay, especially in an industry where the majority of workers are women, have resulted in poor retention, insufficient staffing levels, and limited institutional capacity for change. The neoliberal framing of ECEC as a tool for economic investment, focusing on outcomes such as school readiness and long-term productivity, diminishes its value as a space of care, trust, and protection (Sims, 2017). This perspective devalues the sector’s broader role in supporting children and families, weakening its capacity to prevent and respond to CSA. Safeguarding must be recognised as a core component of quality ECEC, and adequate funding must be allocated to support stable, well-trained workforces capable of implementing best-practice child protection strategies.

#### Perpetrator Accountability for the Abuse of Preverbal and Very Young Children

While the CSA of children under the age of five is not rare, “there is a systematic failure to recognize preverbal CSA as a unique issue” (Jacobson, 2024, p. 448). The majority of protocols or tools to guide investigation of CSA are focused on the child’s ability to disclose in some capacity, and, absent some clear medical sign (such as sexually transmitted infection) or digital evidence (such as CSAM), professionals and courts are provided with little to no guidance on the investigation and prosecution of the abuse of very young children (Jacobson, 2024). Nonetheless, Romano and Hayez (2023) identified a range of definitive and indicative signs of sexual abuse in preverbal children that, they argue, can be used to guide assessment and intervention. There is a clear need for targeted policy and practice development to ensure that offenders who sexually abuse preverbal children are subject to serious criminal penalties, which not only underscores the human rights of babies and toddlers as a particularly vulnerable cohort, but also is likely to have a deterrence effect on offenders who otherwise assume (correctly) that detection and punishment are improbable.

#### Robust Regulation and Enforcement

Finally, robust regulation is essential. Broader concerns have been raised about the oversight of the ECEC sector, as regulatory mechanisms come under pressure from commercial interests and other policy priorities that conflict with the best interests of children (Morrissey & Moore, 2021). The increasing privatisation of the ECEC sector, combined with high demand for childcare places and a focus on cost minimisation in the context of weak and uncoordinated policy, leads to poor-quality ECEC with a range of child safety and wellbeing deficits (Morrissey & Moore, 2021). Child safeguarding in ECEC requires well-resourced and legally empowered regulators to monitor and enforce safeguarding and quality standards, ensuring that information about child maltreatment is transparently gathered and published. Recent legislative updates, such as the strengthening of Reportable Conduct Schemes in Australian states (e.g., Queensland and Victoria) and the implementation of the National Reference System, provide a model for creating centralized oversight bodies capable of tracking allegations across both employers and jurisdictions (National Office for Child Safety, 2025). These measures are critical to closing the information gaps that allowed offenders like Robert M to evade detection across borders. However, the lack of data on CSEA in ECEC identified earlier in this article is not only evidence of a lack of investment in relevant research but, more generally, the lack of data systems to collate and publish information on rates of child abuse and maltreatment in the sector.

## Conclusion

Our case analysis challenges prior claims that ECEC settings are a low risk for CSA or that concerns about child maltreatment in ECEC are a moral panic or otherwise exaggerated. It is perhaps timely to reflect on the legacy and consequences of such claims, which have received broad scholarly and media support in previous decades, given the cumulative evidence of serious and serial CSA offending in ECEC settings. Patterns across the case studies reveal the same institutional weaknesses: failure to act on concerns, over-reliance on external investigations, and a reluctance to question those in trusted roles. The development and evaluation of proactive safeguarding strategies tailored to the unique vulnerabilities of preverbal and very young children in ECEC environments, including the effectiveness of institutional grooming prevention, the promotion of institutional courage and workplace cultures of accountability and transparency, is needed.

This article has highlighted the paucity of research into the prevalence and characteristics of CSA in ECEC. There is a clear need for research into rates of suspected, reported and substantiated CSA cases in ECEC, the response of educators, institutions, regulators and the criminal justice system, and the impact of such abuse upon children and their families. Forensic studies of offenders targeting preverbal children would help to elucidate their specific motivations and strategies.
